# The urban paradox persists: Spatial stationarity in obesity determinants in post-pandemic England

**DOI:** 10.1371/journal.pone.0353465

**Published:** 2026-08-05

**Authors:** Yihan Hu

**Affiliations:** Institute of Metabolic Science Epidemiology Unit, School of Clinical Medicine, University of Cambridge, Cambridge, United Kingdom; Zhejiang University, JAPAN

## Abstract

**Objectives:**

Adult obesity prevalence exhibits significant spatial disparities across England. While traditional global regression models often overlook local variations, it is unclear whether these relationships vary spatially or remain stable. This study aims to robustly identify determinants of clinical obesity and test for spatial non-stationarity using best-practice diagnostics.

**Methods:**

Using 2023/24 data from 151 Upper Tier Local Authorities, we employed a spatial econometric framework. We specified a **row-standardized Queen’s contiguity matrix** for global models. A **Spatial Error Model (SEM)** was benchmarked against the **Spatial Durbin Error Model (SDEM)** to test for the significance of local spillovers, and against Geographically Weighted Regression (GWR) to test for coefficient non-stationarity.

**Results:**

Obesity prevalence showed strong clustering (Moran’s I = 0.58). The SEM (**AICc: 806.4**) significantly outperformed both OLS (AICc: 891.9) and GWR (AICc: 865.3), effectively eliminating residual spatial autocorrelation (Moran’s I = −0.03, *p* > 0.05). Robustness checks using the SDEM did not significantly improve fit (*p* = 0.08). GWR Monte Carlo diagnostics indicated that coefficients for fast-food density and inactivity were spatially stationary (*p* > 0.05). Fast-food density exhibited a robust negative association, supporting the “urban paradox,” while physical inactivity and low fruit/vegetable consumption were significant positive drivers.

**Conclusions:**

Contrary to the “one-size-fits-all” critique, the determinants of obesity appear structurally consistent across England. The “urban paradox” likely reflects broader urbanization patterns rather than direct causality. Policy should focus on national-level structural interventions addressing deprivation and physical activity.

## Introduction

### The post-pandemic context: A syndemic challenge

Obesity remains one of the most entrenched structural challenges facing the United Kingdom. Defined not merely as a health outcome but as a “slow-motion disaster,” it places an unsustainable burden on the National Health Service (NHS), costing an estimated £6.5 billion annually in direct costs alone. However, the landscape of public health has shifted dramatically in the 2020s. The COVID-19 pandemic acted as a “syndemic” event, where biological vulnerability to the virus intersected with, and exacerbated, pre-existing social inequalities [1,2,3].

Furthermore, the subsequent “cost of living” crisis has fundamentally altered household consumption patterns. As food inflation rises, the relative affordability of energy-dense, nutrient-poor foods often increases, potentially deepening the reliance on “cheap calories” [4]. In this volatile post-pandemic context, the government’s “Levelling Up” agenda—aimed at reducing regional disparities—faces a critical test. Understanding the spatial determinants of obesity is no longer merely an epidemiological exercise; it is essential for addressing widening socioeconomic inequalities in the post-pandemic context.

### The policy-evidence mismatch: The “urban paradox”

For decades, the dominant policy narrative has focused on the “obesogenic environment,” specifically the “food swamp” hypothesis. This theory posits a direct causal link between the density of fast-food outlets and obesity prevalence. Guided by this logic, over 50% of Local Authorities in England have adopted planning policies to restrict new hot food takeaways (A5 use class), particularly near schools or in deprived neighborhoods.

However, a critical disconnect has emerged between this policy logic and recent empirical evidence. Large-scale studies have increasingly documented an “urban paradox”: highly urbanized areas, despite having the highest density of fast-food outlets, often exhibit *lower* obesity rates than low-density suburban or rural areas [5]. This counter-intuitive finding suggests that density may act as a proxy for protective “urban advantage” factors—such as walkability, lower car dependency, and younger demographics—rather than a direct risk factor. If this paradox holds true in 2023, current planning restrictions based solely on outlet density may be a “blunt instrument,” misdiagnosing the root causes of the obesity crisis.

### The spatial imperative: Beyond “one-size-fits-all”

The third challenge lies in the methodological rigidity of existing research. Traditional regression models (OLS) assume that the relationship between the environment and health is “stationary”—constant across the entire country. This “one-size-fits-all” assumption violates the basic tenets of geography, ignoring the fact that a fast-food outlet in a walkable London borough may have a different impact than one in a car-dependent town in the North East.

To address this, researchers have increasingly turned to Local models like Geographically Weighted Regression (GWR). While GWR is a powerful tool for visualizing heterogeneity, it has recently come under scrutiny for potential overuse. As noted by [6], there is a tendency to apply local models by default without statistically testing whether the processes are truly non-stationary. This can lead to “false positives,” where random noise is misinterpreted as meaningful spatial variation.

### The obesogenic environment: The “food swamp” hypothesis

The concept of the “obesogenic environment,” first formalized by [7], posits that the dramatic rise in obesity rates cannot be explained solely by individual genetic or psychological shifts. Instead, systemic environmental drivers—specifically the availability of energy-dense food and the layout of the built environment—create a context that promotes weight gain. Within this framework, the availability of fast-food outlets has received extensive scrutiny.

The prevailing theoretical model, often termed the “food swamp” hypothesis, suggests that neighborhoods with a high density of fast-food outlets (relative to healthy options) expose residents to omnipresent cues for unhealthy consumption [8]. Empirical evidence from the UK has historically supported this view. For instance, [9], in a seminal cross-sectional study in Cambridgeshire, found a significant dose-response relationship between takeaway food outlet exposure and body mass index (BMI). These findings have had profound policy implications, leading numerous local authorities in England to implement planning restrictions, such as “exclusion zones” around schools or caps on the density of hot food takeaways (A5 use class) in deprived areas. The logic is linear and intuitive: reducing the density of obesogenic outlets should theoretically reduce consumption and, by extension, obesity prevalence.

### The “urban paradox”: Challenging the density-obesity link

However, the consensus on the “food swamp” effect is far from absolute. As datasets have become more granular and comprehensive, a growing body of literature has identified inconsistencies in the density-obesity relationship. Most notably, [5], utilizing the extensive UK Biobank cohort (*N* > 500,000), identified what has been termed the “urban paradox.” Their analysis revealed that individuals living in areas with the highest density of fast-food outlets—typically dense urban centers—often exhibited *lower* BMI and waist circumference compared to those in low-density areas.

This paradox suggests that fast-food density is not an isolated variable but is deeply confounded by broader urban morphological characteristics. High outlet density often serves as a proxy for “urban advantage” features, such as high intersection density, land-use mix, and destination accessibility, which collectively promote active travel (walking and cycling) [10]. In post-pandemic England, where working patterns and urban mobility have shifted, understanding whether this paradox persists is critical. If high-density areas are protective due to walkability, then planning policies that myopically focus on reducing outlet numbers without addressing the broader built environment may be ineffective or even counterproductive.

### Methodological challenges: From OLS to spatial econometrics

A significant limitation in much of the early literature on obesogenic environments is the reliance on standard Ordinary Least Squares (OLS) regression. OLS assumes that observations are independent of one another, a violation of Tobler’s First Law of Geography: “everything is related to everything else, but near things are more related than distant things” [11].

In the context of obesity, spatial dependence arises from two primary sources:

1. **Spatial Lag (Spillover) Effects:** Obesity behaviors may be socially contagious. Individuals are influenced by the norms and behaviors of their neighbors, suggesting that obesity in one area is functionally related to obesity in adjacent areas.
2. **Spatial Error (Nuisance) Effects:** Unobserved variables—such as local cultural attitudes, micro-climate, or specific local policies—tend to be spatially clustered. If these are omitted from the model, the error terms become spatially correlated, rendering OLS estimates inefficient and hypothesis tests unreliable [12].

Consequently, rigorous analysis requires the adoption of global spatial econometric specifications, such as the Spatial Error Model (SEM) or the Spatial Durbin Model (SDM), which explicitly parameterize these spatial interactions [13,14].

### The stationarity debate: Global vs. local models (GWR)

While global spatial models address autocorrelation, they traditionally assume “spatial stationarity”—that is, the relationship between the environment (e.g., fast food) and the outcome (obesity) is constant across the entire study region. [15] and [16] challenged this assumption, arguing that relationships may vary over space due to contextual heterogeneity or the Modifiable Areal Unit Problem (MAUP) [17]. This critique led to the widespread adoption of Geographically Weighted Regression (GWR) and, more recently, Multi-Scale GWR (MGWR) [18].

However, the rapid popularization of GWR in health geography has drawn methodological criticism. [19] warned that GWR can generate spurious local variations due to local multicollinearity and kernel bandwidth sensitivity, rather than true process heterogeneity. [6] formalized this critique into a “GWR route map,” arguing that local models should not be the default starting point. Instead, researchers must first rigorously test for non-stationarity using Monte Carlo diagnostics. If the regression coefficients do not exhibit statistically significant spatial variation, a properly specified global model (such as the SEM) is theoretically superior due to its parsimony and higher degrees of freedom.

Despite this, few studies on the obesogenic environment explicitly perform this “stationarity test” before interpreting GWR results. This study fills this gap by rigorously benchmarking global versus local specifications to determine whether the determinants of obesity in England are truly spatially heterogeneous or whether they represent a consistent, structural national phenomenon.

### Study objectives

This study seeks to resolve these contradictions by analyzing the determinants of adult obesity across 151 Upper Tier Local Authorities in England using the most recent 2023/24 data. It advances the literature through three specific contributions:

1. **Temporal Update:** It provides the first robust spatial analysis of the “urban paradox” in the post-pandemic, high-inflation era, testing whether previous urban advantages have persisted.
2. **Methodological Rigor:** Unlike studies that arbitrarily choose between global or local models, we employ a rigorous “specification search” strategy. We benchmark the **Spatial Error Model (SEM)** against the **Spatial Durbin Error Model (SDEM)** and **Geographically Weighted Regression (GWR)**, using Monte Carlo diagnostics to definitively test for spatial stationarity.
3. **Policy Recalibration:** By isolating the specific effects of deprivation, inactivity, and food environment density, we aim to provide nuanced evidence to refine the “Levelling Up” health strategy, moving beyond simplistic density caps.

## Materials and methods

### Data sources and processing

This study analyzes spatial data from 151 Upper Tier Local Authorities (UTLAs) in England. The dataset was compiled from the Office for Health Improvement and Disparities (OHID) public health profiles (2023/24). Administrative boundary geometry was obtained from Counties and Unitary Authorities (December 2023) Boundaries UK BFC, provided by the Office for National Statistics Open Geography Portal/data.gov.uk under the Open Government Licence v3.0. The minimal dataset and replication code are publicly available on figshare (https://doi.org/10.6084/m9.figshare.32118484). The dependent variable, *y*, is the prevalence of obesity (BMI ≥ 30). The matrix of explanatory variables, *X*, includes four key determinants selected based on the conceptual framework: Fast Food Density (outlets per 100,000 population), Physical Inactivity (%), Deprivation (IMD Score), and Healthy Eating (5-a-day %).

Prior to spatial modeling, all variables were standardized to facilitate coefficient comparison. We assessed multicollinearity using Variance Inflation Factors (VIF). All VIF values were below 2.5, well below the standard threshold of 10, indicating no severe collinearity issues.

### Spatial weights matrix construction

To quantify spatial relationships, we defined a spatial weights matrix, *W*, based on **Queen’s Contiguity**. Formally, the elements $w_{ij}$ of the matrix are defined as:


$$\begin{array}{c} w_{ij}=\left\{ \begin{array}{cc} 1 & \text{if locations }i\text{ and }j\;\text{share a common boundary or vertex}\\ 0 & \text{otherwise} \end{array}\right. \end{array}$$ (1)

The matrix was **row-standardized** such that $\sum_{j}w_{ij}=1$. This standardization ensures that the spatial lag captures the average value of neighbors, allowing for a meaningful interpretation of spillover parameters.

### Global spatial regression framework

We adopted a “Specific-to-General” specification search strategy [13]. We began with a standard Ordinary Least Squares (OLS) model:


$$\begin{array}{c} y=X\beta +\varepsilon ,\;\varepsilon \sim N(0,\sigma^{2}I) \end{array}$$ (2)

where β is the vector of regression coefficients and ε is the error term.

### Diagnostics and model specification

We employed Lagrange Multiplier (LM) tests to detect spatial dependence. The Robust LM-Error and Robust LM-Lag statistics were compared to distinguish between spatial lag (dependence in the outcome) and spatial error (dependence in omitted variables). Based on the diagnostics (see Results), the **Spatial Error Model (SEM)** was identified as the most appropriate global specification. The SEM accounts for spatial autocorrelation in the error term, formulated as:


$$\begin{array}{cc} y & =X\beta +u\\ u & =\lambda Wu+\varepsilon \end{array}$$ (3)

where λ is the spatial autoregressive coefficient for the error term, measuring the strength of spatial dependence in unobserved factors. *W* is the spatial weights matrix, and ε is the remaining uncorrelated error.

### Robustness check: Spatial Durbin Error Model (SDEM)

To rigorously test whether local spillovers in the covariates were omitted, we nested the SEM within a broader **Spatial Durbin Error Model (SDEM)** [20]. The SDEM adds spatially lagged independent variables (*WX*) to the equation:


$$\begin{array}{cc} y & =X\beta +WX\theta +u\\ u & =\lambda Wu+\varepsilon \end{array}$$ (4)

Here, θ represents the vector of coefficients for the spatially lagged covariates, capturing local spillover effects (e.g., does a neighbor’s deprivation affect focal obesity?). We utilized a **Likelihood Ratio (LR) test** to compare the two models. The test statistic is defined as $LR=-2(L_{SEM}-L_{SDEM})$, following a $\chi^{2}$ distribution with degrees of freedom equal to the number of restrictions (number of covariates in θ). A non-significant LR test (*p* > 0.05) implies that θ=0, justifying the reduction from SDEM to the more parsimonious SEM.

### Local spatial non-stationarity (GWR)

While global models (SEM/SDEM) yield a single coefficient set for the entire study area, Geographically Weighted Regression (GWR) allows coefficients to vary by location. The GWR model is expressed as:


$$\begin{array}{c} y_{i}=\beta_{i0}+\sum_{k}\beta_{ik}x_{ik}+\varepsilon_{i} \end{array}$$ (5)

where $\beta_{ik}$ is the coefficient for variable *k* at location *i*.

To estimate these local parameters, we employed a Weighted Least Squares approach using an **adaptive bi-square kernel** function [21] to determine the spatial weighting scheme. The weight $w_{ij}$ for the kernel is calculated as:


$$\begin{array}{c} w_{ij}=\left\{ \begin{array}{cc} {\left(1-(d_{ij}/b_{i}{)}^{2}\right)}^{2} & \text{if }d_{ij}<b_{i}\\ 0 & \text{otherwise} \end{array}\right. \end{array}$$ (6)

where $d_{ij}$ is the distance between location *i* and *j*, and $b_{i}$ is the adaptive bandwidth (distance to the *n*-th nearest neighbor). The optimal bandwidth was selected by minimizing the Corrected Akaike Information Criterion (AICc) [22].

Crucially, following [23,6], we used GWR primarily as a diagnostic tool. We conducted **Monte Carlo significance tests** (Leung’s F3 test) [24] to formally test the null hypothesis of spatial stationarity for each coefficient. Only if this hypothesis is rejected is the interpretation of local GWR coefficients statistically warranted.

## Results

### Descriptive statistics and spatial structure

The summary statistics for the 151 Upper Tier Local Authorities are presented in Table 1. The mean obesity prevalence is 27.06%, but this masks significant regional disparities, ranging from 11.02% in affluent London boroughs to nearly 38% in post-industrial northern towns.

**Table 1:** Descriptive statistics of variables (*N* = 151).

Variable	Mean	SD	Min	Max	Moran’s I
Obesity Prevalence (%)	27.06	5.84	11.02	37.90	0.58***
Physical Inactivity (%)	22.75	5.27	10.81	36.58	0.31***
Fast Food Density	120.95	39.16	39.16	417.38	0.31***
Deprivation (IMD)	22.96	8.04	5.85	45.04	0.38***
5-a-day Consumption (%)	30.23	5.69	18.84	46.71	0.36***

Note: ***p < 0.001. Dependent variable is defined as adults with **BMI** ≥ **30**. Fast Food Density is measured as outlets per 100,000 population.

Global spatial autocorrelation analysis reveals a profound geographical structure. The Global Moran’s I for obesity is **0.58** (*p* < 0.001), indicating strong positive spatial clustering. This suggests that obesity in England is not randomly distributed but is driven by spatially contiguous structural factors.

**Fig 1:**
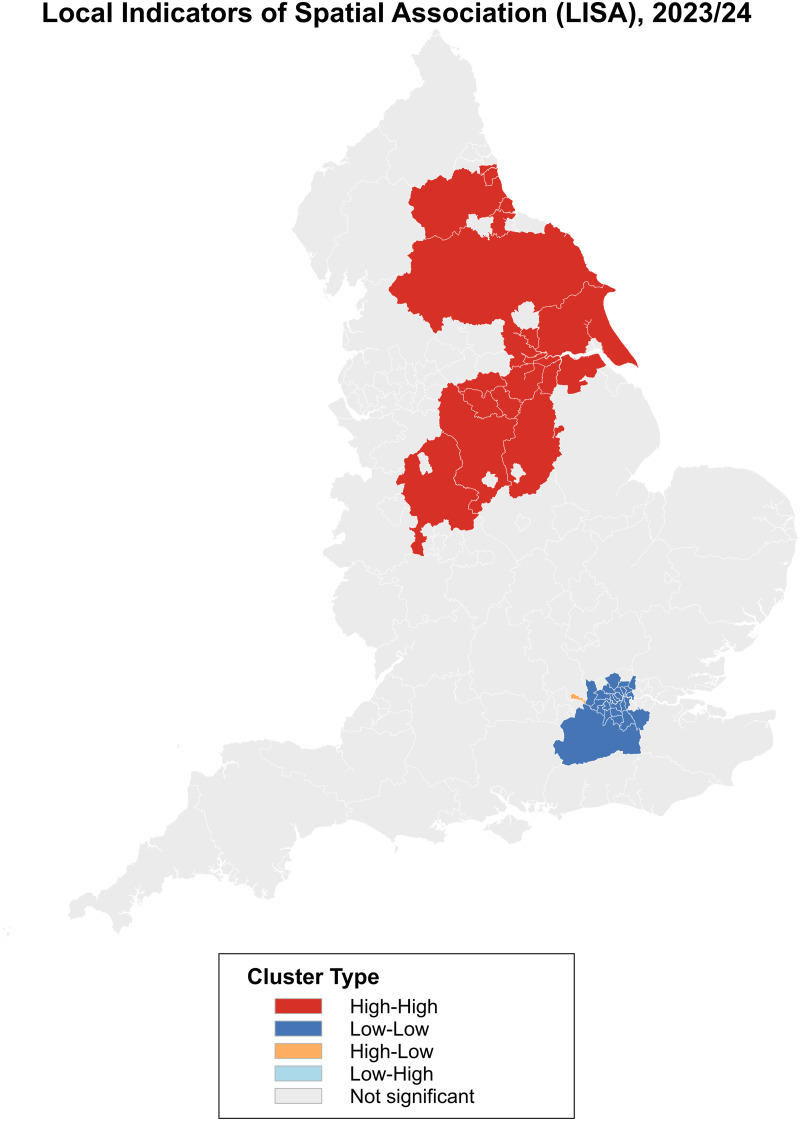
Local Indicators of Spatial Association (LISA) cluster map. Red areas indicate ‘High-High’ clusters (high obesity surrounded by high obesity), significant at *p* < 0.05. Blue areas indicate ‘Low-Low’ clusters (low obesity surrounded by low obesity). This map highlights the structural North-South divide in health outcomes. The figure was generated by the author in R using Counties and Unitary Authorities (December 2023) Boundaries UK BFC from the Office for National Statistics Open Geography Portal/data.gov.uk. Source: Office for National Statistics, licensed under the Open Government Licence v3.0. Contains OS data Crown copyright and database right 2023. No proprietary basemap, satellite imagery, Google Maps, Street View, Mapbox, Esri, or third-party map tiles were used.

**Visualizing the Health Divide:** To decompose this global statistic, the Local Indicators of Spatial Association (LISA) cluster map (Fig 1) visualizes England’s stark “North-South Health Divide.”

- **High-High Clusters (Red):** A large, contiguous corridor of high obesity prevalence dominates the North East, Yorkshire, and the Midlands. These regions historically align with areas of industrial decline and higher deprivation.
- **Low-Low Clusters (Blue):** Conversely, a distinct “cold spot” of obesity is anchored in Greater London and the commuter belt. Crucially, this low-obesity cluster coincides with the areas of highest fast-food density, providing the first spatial signal of the “Urban Paradox.”

**Fig 2:**
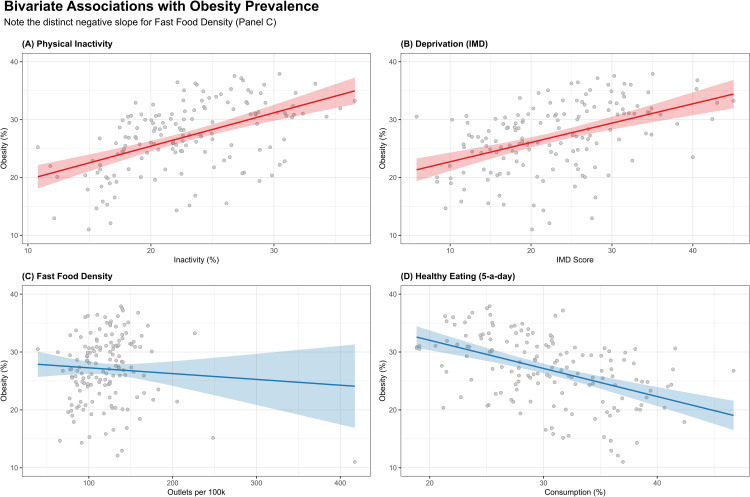
Bivariate associations between key determinants and Obesity Prevalence. Panels (A) and (B) show the expected positive relationships with physical inactivity and deprivation. Panel (C) visually captures the ‘urban paradox,’ illustrating a negative slope between fast-food outlet density and obesity. Panel (D) shows the protective (negative) association with healthy eating.

Before multivariate modeling, we examined bivariate associations (Fig 2). Panels A and B confirm expected positive gradients: inactivity and deprivation are potent drivers of obesity. However, **Panel C visualizes the counter-intuitive ‘Urban Paradox’**. The regression line exhibits a distinct **negative slope**, indicating that UTLAs with the highest density of outlets (often >150 per 100k) tend to have significantly lower obesity rates. This challenges the linear logic of the “food swamp” hypothesis.

### Model selection: Global vs. local specifications

To rigorously determine the appropriate spatial specification, we followed a specific-to-general search strategy. The standard OLS model was rejected due to significant residual spatial autocorrelation (Moran’s I = 0.53, *p* < 0.001), violating the assumption of independent errors. Lagrange Multiplier (LM) diagnostics heavily favored the **Spatial Error** specification (Robust LM-error significant at *p* < 0.01) over the Spatial Lag specification.

We then compared the global **Spatial Error Model (SEM)** against the local **Geographically Weighted Regression (GWR)**.

- **Model Fit (AICc):** The SEM achieved the lowest AICc score (**806.4**), significantly outperforming both OLS (891.9) and GWR (865.3). This suggests that the global model is more parsimonious.
- **Stationarity Test:** Crucially, Monte Carlo stationarity tests (Leung’s F3) on the GWR results revealed **no statistically significant spatial variation** in the coefficients for any covariate (all *p* > 0.05). This indicates that the relationship between the environment and obesity is structurally consistent across England, rendering the complexity of GWR unnecessary.

Finally, we tested the SEM against the more complex **Spatial Durbin Error Model (SDEM)** using a Likelihood Ratio (LR) test. The test failed to reject the null hypothesis ($\chi^{2}(4)=8.27,p=0.08$), confirming that adding local spillover effects (*WX*) did not significantly improve model fit. Thus, the SEM is the robust, preferred specification.

### Determinants of obesity: Analyzing the paradox

The final Spatial Error Model estimates are presented in Table 2. By explicitly modeling the spatial error term (λ=0.76,p<0.001), the SEM effectively eliminated residual autocorrelation (Residual Moran’s I ≈ −0.03).

**Table 2:** Regression Results (Dependent Variable: Obesity Prevalence).

	OLS Model		SEM (Preferred)	
Variable	Coefficient	Std. Error	Coefficient	Std. Error
(Intercept)	24.43***	6.09	28.11***	4.26
Physical Inactivity	0.30*	0.12	0.24***	0.08
Fast Food Density	−0.05***	0.01	−0.02**	0.01
Deprivation (IMD)	0.28***	0.07	0.14**	0.05
5-a-day Consumption	−0.14 (ns)	0.11	−0.21**	0.08
*Spatial Diagnostics*				
Lambda (λ)	–	–	0.76***	0.06
Residual Moran’s I	0.53***		−0.03 (ns)	
**AICc (Standardized)**	891.9		**806.4**	

Note: ***p < 0.001, **p < 0.01, *p < 0.05, ns = not significant.

All Information Criteria are reported as **AICc** to ensure strict comparability between OLS, SEM, and GWR.

**Interpretation of Determinants:**

1. **Structural Dominance of Inactivity:** Physical inactivity remains the strongest positive driver (β=0.24), suggesting that the energy-expenditure side of the equation is critical.
2. **The Paradox Persists:** Even after controlling for deprivation, inactivity, and healthy eating, **Fast Food Density retains a statistically significant negative association** (β=−0.02,p<0.01). This confirms that the “Urban Paradox” is not merely an artifact of omitted variable bias; rather, high-density food environments in England are structurally associated with lower aggregate obesity rates.

**Fig 3:**
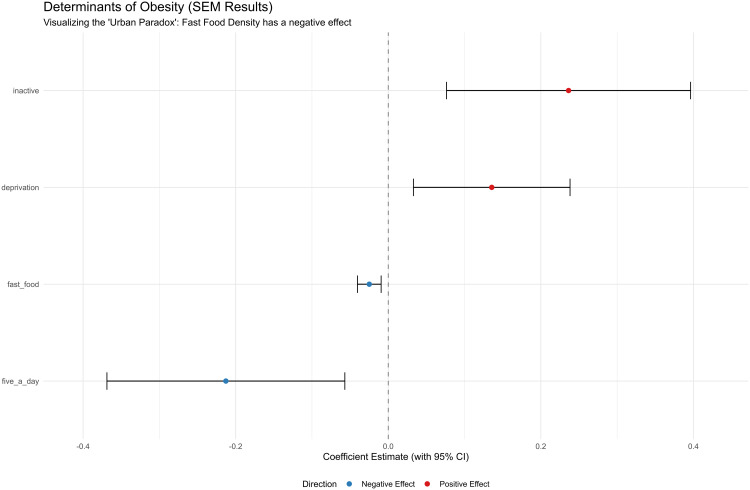
Standardized coefficients from the Spatial Error Model (SEM). Points represent coefficient estimates; bars indicate 95% confidence intervals. Red points indicate positive associations (risk factors), while blue points indicate negative associations. Note the statistically significant negative coefficient for *Fast Food Density*, providing visual confirmation of the ‘urban paradox’.

To visualize these effect sizes, Fig 3 displays the standardized coefficients. The plot starkly highlights the position of Fast Food Density to the left of the zero line (protective), contrasting sharply with the strong risk factors of Deprivation and Inactivity.

## Discussion

### Decoding the ‘urban paradox’: Why density does not equal obesity

The most provocative finding of this study is the robust, statistically significant negative association between fast-food outlet density and obesity prevalence (β=−0.02,p<0.05). This result, visualized in our bivariate and multivariate models, challenges a simple interpretation of the “food swamp” hypothesis. It suggests that, at the UTLA level in post-pandemic England, fast-food outlet density is not a reliable standalone proxy for obesogenic risk.

We propose that this paradox is driven by the **“Urban Advantage”** mechanism. Fast-food outlets do not exist in a vacuum; they cluster in highly accessible, mixed-use urban centers. High outlet density serves as a proxy for a built environment that necessitates and incentivizes **active travel**.

- **Walkability vs. Car Dependency:** Residents in high-density areas (e.g., London boroughs, Manchester city center) are less reliant on private vehicles. The energy expenditure gained from walking to transit hubs, work, or services likely offsets the caloric risk of food accessibility [25,5]. Conversely, low-density suburban areas, while having fewer fast-food outlets, often enforce a sedentary, car-dependent lifestyle, which our model identifies as a stronger driver of obesity (Inactivity β=0.24).
- **Demographic Sorting:** This paradox also reflects demographic sorting. Urban centers with high amenity density attract younger, working-age populations who generally have lower BMI trajectories compared to older populations in semi-rural or suburban belts.

Therefore, the “Food Swamp” is not necessarily a trap; it is often coincident with a “Walkable Haven.”

This finding should not be interpreted as evidence that fast-food consumption is harmless, nor as a direct refutation of neighbourhood-level food-swamp mechanisms. Rather, it helps explain why some smaller-scale studies report positive associations between takeaway exposure and BMI, whereas large-scale urban evidence has identified an inverse or more complex relationship between outlet density and adiposity [9,5]. The apparent contrast likely reflects differences in spatial scale, exposure definition, and confounding by urbanicity.

### Policy implications: From “ban the burger” to “level up”

These findings have profound implications for spatial planning policy in the UK. Currently, over 50% of Local Authorities utilize planning restrictiveness (e.g., banning new A5 takeaways within 400m of schools) as a primary public health lever. Our results suggest that this strategy may be insufficient as a standalone obesity-prevention strategy if it is not accompanied by broader structural interventions.

1. **Misguided Targets:** Since the association between outlet density and obesity is negative (or at best, negligible compared to other factors), restricting the supply of new outlets is unlikely to yield public health dividends. It targets the *symptom* of urban centrality rather than the *cause* of obesity.
2. **The Primacy of Structural Factors:** The dominant coefficients for Physical Inactivity (β=0.24) and Deprivation (β=0.14) indicate that policy should pivot from retail zoning to **structural investment**.
3. **Actionable Recommendations:** Instead of expending resources on complex legal battles to block individual takeaways, planning departments should focus on:
  - **Green Infrastructure:** Investing in parks and active travel corridors to reduce physical inactivity (the strongest predictor).
  - **Poverty Reduction:** Addressing the “cost of living” drivers that force deprived populations toward energy-dense foods, regardless of outlet location.

In the context of the “Levelling Up” agenda, our model confirms that obesity is fundamentally a marker of inequality and inactivity, not merely retail geography. This does not mean that food-environment policy is irrelevant; rather, it suggests that policies focused only on physical outlet counts or zoning may be increasingly incomplete. Recent arguments on ultra-processed food policy similarly emphasise that public health regulation must address the digital food environment as well as the physical streetscape [26].

### Methodological contribution: The case for global structuralism

Beyond the empirical findings, this study offers a critical methodological correction to the recent “local model” turn in health geography. There has been a growing tendency to apply Geographically Weighted Regression (GWR) or MGWR by default, assuming that all health-environment relationships vary over space.

Our rigor in following the **“GWR Route Map”** [6] revealed that such complexity was unwarranted. The Monte Carlo stationarity tests demonstrated that the coefficients for deprivation and food density are statistically stable across England. This is a powerful finding in itself: it suggests that the observed associations are **structural and national**, rather than strongly local and idiosyncratic. In other words, spatial clustering in obesity does not necessarily imply spatial non-stationarity in the effects of measured covariates. By validating the **Spatial Error Model (SEM)**, we demonstrate that “nuisance” spatial dependence (e.g., unmeasured cultural clustering) exists, but the functional relationships between key determinants and health are stationary. This warns against the risk of “overfitting” in spatial epidemiology.

### Limitations

Four limitations should be acknowledged. First, regarding causal inference, the cross-sectional design limits us to identifying associations. Without longitudinal data, we cannot rule out reverse causality, and coefficients should be interpreted as conditional spatial associations rather than strict causal effects [27]. Second, while we employed robust stationarity tests, our measure of “Fast Food Density” is density-based. Neighbourhood outlet density remains useful for characterising physical retail structure, but it increasingly captures only partial exposure in hybrid food systems shaped by delivery platforms, app-based promotion, and digital food marketing [26]. Future research should integrate gravity-based measures and digital food-environment data to capture the nuance of exposure more accurately [28,29]. Third, digital exposure measurement also requires caution. Recent work on TikTok influencer food marketing surveillance emphasises that content prevalence should not be equated with actual adolescent exposure, reinforcing the broader point that availability, visibility, and exposure are distinct constructs in modern food-environment research [30]. Fourth, the “Ecological Fallacy” remains a risk; aggregate UTLA-level relationships may not perfectly mirror individual-level behaviors. Individual-level metabolic heterogeneity, including variation in glycaemic responses to carbohydrates, further cautions against interpreting area-level associations as individual-level mechanisms [31].

## Conclusion

This study revisits the “Urban Paradox” in post-pandemic England, providing robust evidence that the conventional “food swamp” narrative requires revision. Through a rigorous spatial econometric framework, we demonstrate that high fast-food density is associated with *lower* obesity prevalence, likely masking the protective benefits of urban walkability. Crucially, we show that Physical Inactivity and Deprivation are the overwhelming drivers of the spatial patterning of obesity. For policy-makers, the message is clear: the focus must shift from the *restrictive* logic of zoning takeaways to the *enabling* logic of alleviating poverty and building active environments. In a stationary spatial system, structural problems require structural solutions, not local sticking plasters.
