# Supplementary material for: The urban paradox persists: Spatial stationarity in obesity determinants in post-pandemic England
Source: PLoS One. 2026 Aug 5;21(8):e0353465. doi: 10.1371/journal.pone.0353465 (PMC13440795; doi:10.1371/journal.pone.0353465)
Supplement: Supplementary file 1 [file pone.0353465.s002.pdf]

Response to the Academic Editor and Reviewers

Manuscript ID: PONE-D-25-69054

Manuscript title: The urban paradox persists: spatial stationarity in obesity determinants in post-pandemic England

Journal: PLOS ONE

Corresponding author: Yihan Hu

Dear Dr. Ma,

We thank you and the reviewers for the careful evaluation of our manuscript and for the constructive comments. We are pleased that both reviewers found the study technically sound, statistically rigorous, and clearly written. We have revised the manuscript in response to all required and recommended points, while keeping the revisions focused and proportionate to the minor-revision decision.

The main revisions include integrating the literature review and study objectives into the Introduction, strengthening the Discussion through more explicit comparison with existing food-environment and urban-paradox literature, adopting the suggested sentence revision, clarifying the limitations wording, updating the manuscript to the PLOS LaTeX template, depositing the minimal dataset and replication code on figshare, and clarifying the source and licence of the map boundary data used in Figure 1.

Part I. Responses to required and recommended revisions

Comment 1. Improve the structural organisation of the manuscript: integrate research objectives and literature review into the Introduction section.

Response: Thank you for this helpful suggestion. We have revised the manuscript structure so that the literature review is now integrated within the Introduction rather than presented as a separate section. The Introduction now develops the argument in a continuous sequence: the post-pandemic obesity and inequality context, the food-swamp hypothesis, the urban-paradox literature, the spatial econometric and stationarity debate, and finally the study objectives and contributions. The research objectives remain in the Introduction and have been positioned after the relevant literature context.

Changes made: The former Literature Review section has been incorporated into the Introduction, and the subsequent section numbering has been adjusted accordingly.

Comment 2. Strengthen the Discussion section by providing more in-depth comparison and contrast with existing literature.

Response: We agree and have strengthened the Discussion while retaining the original structure of the manuscript. In particular, we added a more explicit comparison between our findings and prior food-swamp studies that reported positive associations between takeaway exposure and BMI at individual or neighbourhood scales. We also clarified that our findings are more consistent with the urban-paradox literature, where dense urban areas may combine high outlet density with protective features such as walkability, public transport accessibility, mixed land use, and younger population structures. We further highlighted that apparent differences across studies may reflect spatial scale, exposure definition, and residual confounding by urbanicity. These additions better contextualise the academic contribution of the study without changing

the core interpretation of the results.

Changes made: The Discussion now more clearly contrasts the present UTLA-level findings with neighbourhood-level food-environment studies and explains how the results contribute to the urban-paradox and spatial-modelling literature.

Comment 3. Revise the key sentence for precise academic expression.

Response: Thank you for the suggested wording. We have adopted the suggested revision. The sentence now reads: "Understanding the spatial determinants of obesity is no longer merely an epidemiological exercise; it is essential for addressing widening socioeconomic inequalities in the post-pandemic context."

Comment 4. Verify the term "several" in the sentence "Several limitations must be acknowledged."

Response: Thank you for noting this. To avoid ambiguity, we have revised the wording. The limitations section now begins: "Four limitations should be acknowledged." We also clarified the limitations relating to cross-sectional design, exposure measurement, ecological inference, and spatial scale.

Comment 5. The manuscript is well written overall, and the above revisions will further enhance its academic rigor and readability.

Response: We thank the reviewer for this encouraging assessment. We have carefully checked the revised manuscript for clarity, consistency, and readability.

Part II. Responses to journal requirements

Journal requirement 1. Ensure that the manuscript meets PLOS ONE style requirements.

Response: We have revised the manuscript to follow PLOS ONE formatting requirements. The title has been converted to sentence case, headings have been adjusted to sentence case where appropriate, figure citations and captions have been checked, and the author affiliation has been updated in the PLOS title/authors/affiliations format.

Journal requirement 2. Update the submission to use the PLOS LaTeX template.

Response: We have migrated the manuscript to the PLOS LaTeX template and used the PLOS bibliography style file for the revised submission.

Journal requirement 3. Ensure that author-generated code is shared without restriction.

Response: All author-generated R code required to reproduce the descriptive statistics, Moran's I statistics, spatial regression models, LISA cluster analysis, and figures has been deposited on figshare without restriction, together with the minimal dataset and metadata.

Journal requirement 4. Deposit the minimal dataset in an acceptable public repository.

Response: We have deposited the minimal dataset and replication code in figshare, an accepted stable public repository. The DOI is: `https://doi.org/10.6084/m9.figshare.32118484`. The repository contains the area-level public health indicators, spatial boundary data, metadata, and R code necessary to reproduce the results reported in the manuscript.

Journal requirement 5. Ensure that the abstract in the online submission form and manuscript are identical.

Response: We will ensure that the abstract entered in the Editorial Manager submission form is identical to the abstract in the revised manuscript.

Journal requirement 6. Clarify copyright and licence status for Figure 1.

Response: Thank you for highlighting the copyright and licence requirements for map figures. Figure 1 was generated by the author in R using Counties and Unitary Authorities (December 2023) Boundaries UK BFC from the Office for National Statistics Open Geography Portal/data.gov.uk. The boundary data are licensed under the Open Government Licence v3.0, and the required attribution to the Office for National Statistics and Ordnance Survey data has been added to the revised figure caption. The figure does not contain any proprietary basemap, satellite imagery, Google Maps, Street View, Mapbox, Esri, or third-party map tiles. We have revised the Figure 1 caption to specify the data source, licence, and attribution information.

Journal requirement 7. Upload new copies of Figures 2 and 3 because the detail was not clear.

Response: We have prepared and uploaded new higher-resolution versions of Figures 2 and 3. The revised figures use larger text, clearer axis labels, and improved export settings to enhance readability. We also re-exported Figure 1 for consistency.

Journal requirement 8. Review any recommended previously published works and cite them where relevant.

Response: No specific mandatory references were requested by the reviewers. Therefore, no action was required for this item. We nevertheless ensured that all newly added citations are directly relevant to the revised Discussion and limitations.

Journal requirement 9. Review the reference list for completeness, correctness, and retracted articles.

Response: We have reviewed the reference list for completeness and accuracy. To the best of our knowledge, the manuscript does not cite any retracted articles. We also note that one newly cited Lancet Correspondence is currently accepted/in press and expected to appear online shortly. At the time of resubmission it is cited as an in-press article; we will update the reference with final bibliographic details during proof correction if these become available.

Closing statement

We believe these revisions address all points raised by the Academic Editor, reviewers, and journal office. We thank you again for the opportunity to revise the manuscript and hope that the revised version is now suitable for publication in PLOS ONE.

Sincerely,

Yihan Hu

Corresponding author
