# Supplementary material for: The urban paradox persists: Spatial stationarity in obesity determinants in post-pandemic England
Source: PLoS One. 2026 Aug 5;21(8):e0353465. doi: 10.1371/journal.pone.0353465 (PMC13440795; doi:10.1371/journal.pone.0353465)
Supplement: Peer review history

# Peer review: Decision Letter 0

**DOI:** 10.1371/journal.pone.0353465.r001

**Authors:** Shuang Ma

28 Apr 2026

PONE-D-25-69054

The Urban Paradox Persists: Spatial Stationarity in Obesity Determinants in Post-Pandemic England

PLOS One

Dear Dr. Hu,

Thank you for submitting your manuscript to PLOS ONE. After careful consideration, we feel that it has merit but does not fully meet PLOS ONE’s publication criteria as it currently stands. Therefore, we invite you to submit a revised version of the manuscript that addresses the points raised during the review process.

==============================

Based on the peer review comments and PLOS ONE’s publication criteria, the following revisions are required for acceptance:

Required Revisions (Must Address for Acceptance)

1. Improve the structural organisation of the manuscript: Integrate research objectives and literature review into the Introduction section.

2. Strengthen the Discussion section: Provide in-depth comparison and contrast with existing literature to highlight the contextual relevance and academic contribution of your findings.

Recommended Revisions (Suggested for Improvement)

3. Revise the key sentence for precise academic expression: Consider adopting the alternative version: “Understanding the spatial determinants of obesity is no longer merely an epidemiological exercise; it is essential for addressing widening socioeconomic inequalities in the post-pandemic period / post-pandemic context.”

4. Verify the term “several” in the sentence “Several limitations must be acknowledged”: Confirm whether 2–3 limitations align with the connotation of “several” and adjust the expression if necessary.

5. The manuscript is well written overall, and the above revisions will further enhance its academic rigor and readability.

==============================

Please submit your revised manuscript by Jun 12 2026 11:59PM. If you will need more time than this to complete your revisions, please reply to this message or contact the journal office at plosone@plos.org. When you're ready to submit your revision, log on to https://www.editorialmanager.com/pone/ and select the 'Submissions Needing Revision' folder to locate your manuscript file.

Please include the following items when submitting your revised manuscript:

- A letter that responds to each point raised by the academic editor and reviewer(s). You should upload this letter as a separate file labeled 'Response to Reviewers'.
- A marked-up copy of your manuscript that highlights changes made to the original version. You should upload this as a separate file labeled 'Revised Manuscript with Track Changes'.
- An unmarked version of your revised paper without tracked changes. You should upload this as a separate file labeled 'Manuscript'.

If you would like to make changes to your financial disclosure, please include your updated statement in your cover letter. Guidelines for resubmitting your figure files are available below the reviewer comments at the end of this letter.

If applicable, we recommend that you deposit your laboratory protocols in protocols.io to enhance the reproducibility of your results. Protocols.io assigns your protocol its own identifier (DOI) so that it can be cited independently in the future. For instructions see: https://journals.plos.org/plosone/s/submission-guidelines#loc-laboratory-protocols. Additionally, PLOS ONE offers an option for publishing peer-reviewed Lab Protocol articles, which describe protocols hosted on protocols.io. Read more information on sharing protocols at https://plos.org/protocols?utm_medium=editorial-email&utm_source=authorletters&utm_campaign=protocols.

As the corresponding author, your ORCID iD is verified in the submission system and will appear in the published article. PLOS supports the use of ORCID, and we encourage all coauthors to register for an ORCID iD and use it as well. Please encourage your coauthors to verify their ORCID iD within the submission system before final acceptance, as unverified ORCID iDs will not appear in the published article. *Only*  the individual author can complete the verification step; PLOS staff *cannot*  verify ORCID iDs on behalf of authors.

We look forward to receiving your revised manuscript.

Kind regards,

Shuang Ma

Academic Editor

PLOS One

**Journal Requirements:**

1. When submitting your revision, we need you to address these additional requirements.

Please ensure that your manuscript meets PLOS ONE's style requirements, including those for file naming. The PLOS ONE style templates can be found at

https://journals.plos.org/plosone/s/file?id=wjVg/PLOSOne_formatting_sample_main_body.pdf and

https://journals.plos.org/plosone/s/file?id=ba62/PLOSOne_formatting_sample_title_authors_affiliations.pdf

2. Please update your submission to use the PLOS LaTeX template. The template and more information on our requirements for LaTeX submissions can be found at http://journals.plos.org/plosone/s/latex.

3. Please note that PLOS One has specific guidelines on code sharing for submissions in which author-generated code underpins the findings in the manuscript. In these cases, we expect all author-generated code to be made available without restrictions upon publication of the work. Please review our guidelines at https://journals.plos.org/plosone/s/materials-and-software-sharing#loc-sharing-code and ensure that your code is shared in a way that follows best practice and facilitates reproducibility and reuse.

4. Thank you for uploading your study's underlying data set. Unfortunately, the repository you have noted in your Data Availability statement does not qualify as an acceptable data repository according to PLOS's standards.

At this time, please upload the minimal data set necessary to replicate your study's findings to a stable, public repository (such as figshare or Dryad) and provide us with the relevant URLs, DOIs, or accession numbers that may be used to access these data. For a list of recommended repositories and additional information on PLOS standards for data deposition, please see https://journals.plos.org/plosone/s/recommended-repositories.

5. Please amend either the abstract on the online submission form (via Edit Submission) or the abstract in the manuscript so that they are identical.

6. We note that Figure 1 in your submission contain map images which may be copyrighted. All PLOS content is published under the Creative Commons Attribution License (CC BY 4.0), which means that the manuscript, images, and Supporting Information files will be freely available online, and any third party is permitted to access, download, copy, distribute, and use these materials in any way, even commercially, with proper attribution. For these reasons, we cannot publish previously copyrighted maps or satellite images created using proprietary data, such as Google software (Google Maps, Street View, and Earth). For more information, see our copyright guidelines: http://journals.plos.org/plosone/s/licenses-and-copyright.

We require you to either (present written permission from the copyright holder to publish these figures specifically under the CC BY 4.0 license, or remove the figures from your submission:

a. You may seek permission from the original copyright holder of Figure 1 to publish the content specifically under the CC BY 4.0 license.

We recommend that you contact the original copyright holder with the Content Permission Form (http://journals.plos.org/plosone/s/file?id=7c09/content-permission-form.pdf) and the following text:

“I request permission for the open-access journal PLOS ONE to publish XXX under the Creative Commons Attribution License (CCAL) CC BY 4.0 (http://creativecommons.org/licenses/by/4.0/). Please be aware that this license allows unrestricted use and distribution, even commercially, by third parties. Please reply and provide explicit written permission to publish XXX under a CC BY license and complete the attached form.”

Please upload the completed Content Permission Form or other proof of granted permissions as an "Other" file with your submission.

In the figure caption of the copyrighted figure, please include the following text: “Reprinted from [ref] under a CC BY license, with permission from [name of publisher], original copyright [original copyright year].”

b. If you are unable to obtain permission from the original copyright holder to publish these figures under the CC BY 4.0 license or if the copyright holder’s requirements are incompatible with the CC BY 4.0 license, please either i) remove the figure or ii) supply a replacement figure that complies with the CC BY 4.0 license. Please check copyright information on all replacement figures and update the figure caption with source information. If applicable, please specify in the figure caption text when a figure is similar but not identical to the original image and is therefore for illustrative purposes only.

The following resources for replacing copyrighted map figures may be helpful:

USGS National Map Viewer (public domain): http://viewer.nationalmap.gov/viewer/

The Gateway to Astronaut Photography of Earth (public domain): http://eol.jsc.nasa.gov/sseop/clickmap/

Maps at the CIA (public domain): https://www.cia.gov/library/publications/the-world-factbook/index.html and https://www.cia.gov/library/publications/cia-maps-publications/index.html

NASA Earth Observatory (public domain): http://earthobservatory.nasa.gov/

Landsat: http://landsat.visibleearth.nasa.gov/

USGS EROS (Earth Resources Observatory and Science (EROS) Center) (public domain): http://eros.usgs.gov/#

Natural Earth (public domain): http://www.naturalearthdata.com/

7. Please upload a new copy of Figures 2 and 3, as the detail is not clear. Please follow the link for more information:  https://journals.plos.org/plosone/s/figures

8. If the reviewer comments include a recommendation to cite specific previously published works, please review and evaluate these publications to determine whether they are relevant and should be cited. There is no requirement to cite these works unless the editor has indicated otherwise.

9. Please review your reference list to ensure that it is complete and correct. If you have cited papers that have been retracted, please include the rationale for doing so in the manuscript text, or remove these references and replace them with relevant current references. Any changes to the reference list should be mentioned in the rebuttal letter that accompanies your revised manuscript. If you need to cite a retracted article, indicate the article’s retracted status in the References list and also include a citation and full reference for the retraction notice.

**Additional Editor Comments:**

Two reviewers have evaluated your manuscript. One reviewer recommends Accept with only minor wording adjustments; the other reviewer suggests Minor Revision with structural and content improvement requirements. The detailed consolidated comments are listed below:

1. Revise the key sentence for accuracy and academic expression, for example, Understanding the spatial determinants of obesity is no longer merely an epidemiological exercise; it is essential for addressing widening socioeconomic inequalities in the post-pandemic period / post-pandemic context.

2. Re-evaluate the use of the term “several” in the sentence “Several limitations must be acknowledged”. Please check whether 2–3 limitations are suitable for this expression and revise if needed.

3. The manuscript is well written, and the overall quality is highly recognised.

4. Improve the structural organisation of the manuscript. Research objectives and literature review should be properly integrated into the Introduction section.

5. Further strengthen the Discussion section. Provide more in-depth comparison and contrast with existing literature, to better highlight the contribution and contextual relevance of your research findings.

[Note: HTML markup is below. Please do not edit.]

Reviewers' comments:

Reviewer's Responses to Questions

**Comments to the Author**

1. Is the manuscript technically sound, and do the data support the conclusions?

The manuscript must describe a technically sound piece of scientific research with data that supports the conclusions. Experiments must have been conducted rigorously, with appropriate controls, replication, and sample sizes. The conclusions must be drawn appropriately based on the data presented.

Reviewer #1: Yes

Reviewer #2: Yes

**********

2. Has the statistical analysis been performed appropriately and rigorously?

Reviewer #1: Yes

Reviewer #2: Yes

**********

3. Have the authors made all data underlying the findings in their manuscript fully available?

The PLOS Data policy requires authors to make all data underlying the findings described in their manuscript fully available without restriction, with rare exception (please refer to the Data Availability Statement in the manuscript PDF file). The data should be provided as part of the manuscript or its supporting information, or deposited to a public repository. For example, in addition to summary statistics, the data points behind means, medians and variance measures should be available. If there are restrictions on publicly sharing data—e.g. participant privacy or use of data from a third party—those must be specified.

Reviewer #1: Yes

Reviewer #2: Yes

**********

4. Is the manuscript presented in an intelligible fashion and written in standard English?

PLOS ONE does not copyedit accepted manuscripts, so the language in submitted articles must be clear, correct, and unambiguous. Any typographical or grammatical errors should be corrected at revision, so please note any specific errors here.

Reviewer #1: Yes

Reviewer #2: Yes

**********

5. Review Comments to the Author

Please use the space provided to explain your answers to the questions above. You may also include additional comments for the author, including concerns about dual publication, research ethics, or publication ethics. (Please upload your review as an attachment if it exceeds 20,000 characters)

**Reviewer #1:** Commendation to the authors for the job well

"Furthermore, the subsequent "cost of living" crisis has fundamentally altered household con3

5 sumption patterns. As food inflation rises, the relative affordability of energy-dense, nutrient36 poor foods often increases, potentially deepening the reliance on "cheap calories" (Dimbleby, 37 2021). In this volatile post-pandemic context, the government’s "Levelling Up" agenda—aimed

38 at reducing regional disparities—faces a critical test. Understanding the spatial determinants of

39 obesity is no longer just an epidemiological exercise; it is a prerequisite for effective economic

40 and social recovery"

i would prefer using " Understanding the spatial determinants of obesity is no longer merely an epidemiological exercise; it is essential for addressing widening socioeconomic inequalities in the post-pandemic period OR post-pandemic context."

Also, "Several limitations must be acknowledged. " Does 2 or 3 qualify as several?

Well written

**Reviewer #2:**  The study addresses an important health issue.

Comments:

1. The manuscript requires better structural organisation. Objectives and literature review should be included within the introduction section.

2. The discussion section needs to be strengthened. It should include more thorough comparison and contrast with existing studies to contextualise the relevance of the findings.

**********

6. PLOS authors have the option to publish the peer review history of their article (what does this mean?). If published, this will include your full peer review and any attached files.

If you choose “no”, your identity will remain anonymous but your review may still be made public.

**Do you want your identity to be public for this peer review?**  For information about this choice, including consent withdrawal, please see our Privacy Policy.

Reviewer #1: No

Reviewer #2: No

**********

[NOTE: If reviewer comments were submitted as an attachment file, they will be attached to this email and accessible via the submission site. Please log into your account, locate the manuscript record, and check for the action link "View Attachments". If this link does not appear, there are no attachment files.]

To ensure your figures meet our technical requirements, please review our figure guidelines: https://journals.plos.org/plosone/s/figures

You may also use PLOS’s free figure tool, NAAS, to help you prepare publication quality figures: https://journals.plos.org/plosone/s/figures#loc-tools-for-figure-preparation.

NAAS will assess whether your figures meet our technical requirements by comparing each figure against our figure specifications.

# Author comment: Author response to Decision Letter 1

**DOI:** 10.1371/journal.pone.0353465.r002

4 May 2026

Response to the Academic Editor and Reviewers

Manuscript ID: PONE-D-25-69054

Title: The urban paradox persists: spatial stationarity in obesity determinants in post-pandemic England

Dear Dr. Ma and Reviewers,

Thank you for the careful evaluation of our manuscript and for the constructive comments. We are pleased that both reviewers found the study technically sound, statistically rigorous, and clearly written. We have revised the manuscript in response to all required and recommended points, while keeping the revisions focused and proportionate to the minor-revision decision.

The main revisions include integrating the literature review and study objectives into the Introduction, strengthening the Discussion through more explicit comparison with existing food-environment and urban-paradox literature, revising the suggested sentence, clarifying the wording of the limitations section, updating the manuscript to the PLOS LaTeX template, depositing the minimal dataset and replication code on figshare, and clarifying the source and licence information for the map boundary data used in Figure 1.

Responses to the Academic Editor and Reviewers

Comment 1: Improve the structural organisation of the manuscript: integrate research objectives and literature review into the Introduction section.

Response: Thank you for this helpful suggestion. We have revised the manuscript structure so that the literature review is now integrated within the Introduction rather than presented as a separate section. The Introduction now develops the argument in a continuous sequence: the post-pandemic obesity and inequality context, the food-swamp hypothesis, the urban-paradox literature, the spatial econometric and stationarity debate, and finally the study objectives and contributions. The research objectives remain in the Introduction and have been positioned after the relevant literature context.

Comment 2: Strengthen the Discussion section by providing more in-depth comparison and contrast with existing literature.

Response: We agree and have strengthened the Discussion while retaining the original structure of the manuscript. In particular, we added a more explicit comparison between our findings and prior food-swamp studies that reported positive associations between takeaway exposure and BMI at individual or neighbourhood scales. We also clarified that our findings are more consistent with the urban-paradox literature, where dense urban areas may combine high outlet density with protective features such as walkability, public transport accessibility, mixed land use, and younger population structures. We further highlighted that apparent differences across studies may reflect spatial scale, exposure definition, and residual confounding by urbanicity. These additions better contextualise the academic contribution of the study without changing the core interpretation of the results.

Comment 3: Revise the key sentence for precise academic expression.

Response: Thank you for the suggested wording. We have adopted the suggested revision. The sentence now reads: “Understanding the spatial determinants of obesity is no longer merely an epidemiological exercise; it is essential for addressing widening socioeconomic inequalities in the post-pandemic context.”

Comment 4: Verify the term “several” in the sentence “Several limitations must be acknowledged.”

Response: Thank you for noting this. To avoid ambiguity, we have revised the wording. The limitations section now begins: “Four limitations should be acknowledged.” We also clarified the limitations relating to cross-sectional design, exposure measurement, ecological inference, and spatial scale.

Comment 5: The manuscript is well written overall, and the above revisions will further enhance its academic rigor and readability.

Response: We thank the reviewer for this encouraging assessment. We have carefully checked the revised manuscript for clarity, consistency, and readability.

Responses to Journal Requirements

Journal requirement 1: Ensure that the manuscript meets PLOS ONE style requirements.

Response: We have revised the manuscript to follow PLOS ONE formatting requirements. The title has been converted to sentence case, headings have been adjusted to sentence case where appropriate, figure citations and captions have been checked, and the author affiliation has been updated in the PLOS title/authors/affiliations format.

Journal requirement 2: Update the submission to use the PLOS LaTeX template.

Response: We have migrated the manuscript to the PLOS LaTeX template and used the PLOS bibliography style file for the revised submission.

Journal requirement 3: Ensure that author-generated code is shared without restriction.

Response: All author-generated R code required to reproduce the descriptive statistics, Moran’s I statistics, spatial regression models, LISA cluster analysis, and figures has been deposited on figshare without restriction, together with the minimal dataset and metadata.

Journal requirement 4: Deposit the minimal dataset in an acceptable public repository.

Response: We have deposited the minimal dataset and replication code in figshare, an accepted stable public repository. The DOI is: https://doi.org/10.6084/m9.figshare.32118484. The repository contains the area-level public health indicators, spatial boundary data, metadata, and R code necessary to reproduce the results reported in the manuscript.

Journal requirement 5: Ensure that the abstract in the online submission form and manuscript are identical.

Response: We have ensured that the abstract entered in the Editorial Manager submission form is identical to the abstract in the revised manuscript.

Journal requirement 6: Clarify copyright and licence status for Figure 1.

Response: Thank you for highlighting the copyright and licence requirements for map figures. Figure 1 was generated by the author in R using Counties and Unitary Authorities (December 2023) Boundaries UK BFC from the Office for National Statistics Open Geography Portal/data.gov.uk. The boundary data are licensed under the Open Government Licence v3.0, and the required attribution to the Office for National Statistics and Ordnance Survey data has been added to the revised figure caption. The figure does not contain any proprietary basemap, satellite imagery, Google Maps, Street View, Mapbox, Esri, or third-party map tiles. We have revised the Figure 1 caption to specify the data source, licence, and attribution information.

Journal requirement 7: Upload new copies of Figures 2 and 3 because the detail was not clear.

Response: We have prepared and uploaded new higher-resolution versions of Figures 2 and 3. The revised figures use clearer export settings to enhance readability. We also re-exported Figure 1 for consistency.

Journal requirement 8: Review any recommended previously published works and cite them where relevant.

Response: No specific mandatory references were requested by the reviewers. We have therefore not treated any additional citation as required. We nevertheless reviewed the revised manuscript to ensure that all added citations are directly relevant to the strengthened Discussion and limitations.

Journal requirement 9: Review the reference list for completeness, correctness, and retracted articles.

Response: We have reviewed the reference list for completeness and accuracy. To the best of our knowledge, the manuscript does not cite any retracted articles. One accepted Lancet Correspondence has been cited as “in press”; as it is expected to be available online shortly, the final bibliographic details will be updated during proofreading or final bibliographic checking once the official online record is available.

We believe these revisions address all points raised by the Academic Editor, reviewers, and journal office. We thank you again for the opportunity to revise the manuscript and hope that the revised version is now suitable for publication in PLOS ONE.

Sincerely,

Yihan Hu

Corresponding author

Attachment Submitted filename: Response_to_Reviewers_PONE-D-25-69054.pdf

# Peer review: Decision Letter 1

**DOI:** 10.1371/journal.pone.0353465.r003

**Authors:** Shuang Ma, Shuang Ma

24 Jun 2026

The Urban Paradox Persists: Spatial Stationarity in Obesity Determinants in Post-Pandemic England

PONE-D-25-69054R1

Dear Dr. Hu,

We’re pleased to inform you that your manuscript has been judged scientifically suitable for publication and will be formally accepted for publication once it meets all outstanding technical requirements.

Within one week, you’ll receive an e-mail detailing the required amendments. When these have been addressed, you’ll receive a formal acceptance letter and your manuscript will be scheduled for publication.

An invoice will be generated when your article is formally accepted. Please note, if your institution has a publishing partnership with PLOS and your article meets the relevant criteria, all or part of your publication costs will be covered. Please make sure your user information is up-to-date by logging into Editorial Manager at Editorial Manager® and clicking the ‘Update My Information' link at the top of the page. For questions related to billing, please contact billing support.

If your institution or institutions have a press office, please notify them about your upcoming paper to help maximize its impact. If they’ll be preparing press materials, please inform our press team as soon as possible -- no later than 48 hours after receiving the formal acceptance. Your manuscript will remain under strict press embargo until 2 pm Eastern Time on the date of publication. For more information, please contact onepress@plos.org.

Kind regards,

Shuang Ma

Academic Editor

PLOS One

Additional Editor Comments (optional):

Reviewers' comments:

Reviewer's Responses to Questions

**Comments to the Author**

1. If the authors have adequately addressed your comments raised in a previous round of review and you feel that this manuscript is now acceptable for publication, you may indicate that here to bypass the “Comments to the Author” section, enter your conflict of interest statement in the “Confidential to Editor” section, and submit your "Accept" recommendation.

Reviewer #2: All comments have been addressed

**********

2. Is the manuscript technically sound, and do the data support the conclusions?

The manuscript must describe a technically sound piece of scientific research with data that supports the conclusions. Experiments must have been conducted rigorously, with appropriate controls, replication, and sample sizes. The conclusions must be drawn appropriately based on the data presented.

Reviewer #2: Yes

**********

3. Has the statistical analysis been performed appropriately and rigorously?

Reviewer #2: Yes

**********

4. Have the authors made all data underlying the findings in their manuscript fully available?

The PLOS Data policy requires authors to make all data underlying the findings described in their manuscript fully available without restriction, with rare exception (please refer to the Data Availability Statement in the manuscript PDF file). The data should be provided as part of the manuscript or its supporting information, or deposited to a public repository. For example, in addition to summary statistics, the data points behind means, medians and variance measures should be available. If there are restrictions on publicly sharing data—e.g. participant privacy or use of data from a third party—those must be specified.

Reviewer #2: Yes

**********

5. Is the manuscript presented in an intelligible fashion and written in standard English?

PLOS ONE does not copyedit accepted manuscripts, so the language in submitted articles must be clear, correct, and unambiguous. Any typographical or grammatical errors should be corrected at revision, so please note any specific errors here.

Reviewer #2: Yes

**********

6. Review Comments to the Author

Please use the space provided to explain your answers to the questions above. You may also include additional comments for the author, including concerns about dual publication, research ethics, or publication ethics. (Please upload your review as an attachment if it exceeds 20,000 characters)

Reviewer #2: (No Response)

**********

7. PLOS authors have the option to publish the peer review history of their article (what does this mean?). If published, this will include your full peer review and any attached files.

If you choose “no”, your identity will remain anonymous but your review may still be made public.

**Do you want your identity to be public for this peer review?**  For information about this choice, including consent withdrawal, please see our Privacy Policy.

Reviewer #2: No

**********

# Editor report: Acceptance letter

**DOI:** 10.1371/journal.pone.0353465.r004

**Authors:** Shuang Ma, Shuang Ma

PONE-D-25-69054R1

PLOS One

Dear Dr. Hu,

I'm pleased to inform you that your manuscript has been deemed suitable for publication in PLOS One. Congratulations! Your manuscript is now being handed over to our production team.

At this stage, our production department will prepare your paper for publication. This includes ensuring the following:

* All references, tables, and figures are properly cited

* All relevant supporting information is included in the manuscript submission,

* There are no issues that prevent the paper from being properly typeset

You will receive further instructions from the production team, including instructions on how to review your proof when it is ready. Please keep in mind that we are working through a large volume of accepted articles, so please give us a few days to review your paper and let you know the next and final steps.

Lastly, if your institution or institutions have a press office, please let them know about your upcoming paper now to help maximize its impact. If they'll be preparing press materials, please inform our press team within the next 48 hours. Your manuscript will remain under strict press embargo until 2 pm Eastern Time on the date of publication. For more information, please contact onepress@plos.org.

You will receive an invoice from PLOS for your publication fee after your manuscript has reached the completed accept phase. If you receive an email requesting payment before acceptance or for any other service, this may be a phishing scheme. Learn how to identify phishing emails and protect your accounts at https://explore.plos.org/phishing.

If we can help with anything else, please email us at customercare@plos.org.

Thank you for submitting your work to PLOS ONE and supporting open access.

Kind regards,

PLOS ONE Editorial Office Staff

on behalf of

Dr. Shuang Ma

Academic Editor

PLOS One
